# c-FLIP is involved in tumor progression of peripheral T-cell lymphoma and targeted by histone deacetylase inhibitors

**DOI:** 10.1186/s13045-014-0088-y

**Published:** 2014-12-05

**Authors:** Zhong Zheng, Shu Cheng, Wen Wu, Li Wang, Yan Zhao, Yang Shen, Anne Janin, Wei-Li Zhao

**Affiliations:** State Key Laboratory of Medical Genomics, Shanghai Institute of Hematology, Shanghai Rui Jin Hospital, Shanghai Jiao Tong University School of Medicine, 197 Rui Jin Er Road, Shanghai, 200025 China; Pôle de Recherches Sino-Français en Science du Vivant et Génomique, Laboratory of Molecular Pathology, Shanghai, China; U1165 Inserm/Université Paris 7, Hôpital Saint Louis, Pairs, France

**Keywords:** Peripheral T-cell lymphoma, Apoptosis, c-FLIP, Histone deacetylase inhibitor, NF-κB

## Abstract

**Background:**

Peripheral T-cell lymphomas (PTCLs) are often aggressive tumors and resistant to conventional chemotherapy. Dysregulation of extrinsic apoptosis plays an important role on tumor cell sensitivity to chemotherapeutic agents. Cellular FLICE inhibitory protein (c-FLIP) is a key regulator of extrinsic apoptotic pathway.

**Methods:**

*c-FLIP* expression was assessed by real-time PCR and compared according to clinical parameters in patients with PTCLs. The relation of c-FLIP to tumor cell apoptosis mediated by histone deacetylases inhibitors (HDACIs) and the possible mechanism were examined in T-lymphoma cell lines and in a murine xenograft model.

**Results:**

*c-FLIP* was overexpressed and associated with decreased tumor TRAIL/DR5 expression, elevated serum lactate dehydrogenase level and high-risk International Prognostic Index of the patients. In vitro, molecular silencing of *c-FLIP* by specific small-interfering RNA increased TRAIL/DR5 expression, enhanced T-lymphoma cell apoptosis and sensitized cells to chemotherapeutic agents. However, HDACIs valproic acid (VPA) and suberoylanilide hydroxamic acid (SAHA) could downregulate c-FLIP expression and triggered extrinsic apoptosis of T-lymphoma cells, through inhibiting NF-κB signaling and interrupting P50 interaction with c-FLIP promoter. As Class I HDACIs, both VPA and SAHA inhibited HDAC1, resulting in P50 inactivation and c-FLIP downregulation. In vivo, oral VPA treatment significantly retarded tumor growth and induced in situ apoptosis, consistent with inhibition of HDAC1/P50/c-FLIP axis and increase of TRAIL/DR5 expression.

**Conclusions:**

c-FLIP overexpression in PTCLs protected tumor cells from extrinsic apoptosis and contributed to tumor progression. Although linking to chemoresistance, c-FLIP indicated tumor cell sensitivity to HDACIs, providing a potential biomarker of targeting apoptosis in treating PTCLs.

**Electronic supplementary material:**

The online version of this article (doi:10.1186/s13045-014-0088-y) contains supplementary material, which is available to authorized users.

## Background

Peripheral T-cell lymphomas (PTCLs) are derived from malignant proliferation of mature T-lymphocytes and represent 10%-15% of non-Hodgkin’s lymphomas [1,2]. Compared with B-cell lymphomas, PTCLs are often aggressive and have inferior disease outcome with current treatment paradigms. Thus, new bio-therapeutic agents should be identified to further improve the prognosis of PTCL patients.

Dysregulation of apoptosis is generally implicated in tumor progression [3,4]. While anti-apoptotic genes of the intrinsic pathway like *BCL-2* and *BCL-XL* are constitutively activated in their B-cell counterparts [5], T-cell lymphomas are frequently present with defect in extrinsic apoptosis. Cellular FLICE inhibitory protein (c-FLIP) is a key regulator of extrinsic apoptotic signaling and induces resistance to death receptor-mediated apoptosis [6]. c-FLIP is overexpressed in tumors of various origins including non-Hodgkin’s lymphoma and correlated with poor clinical outcome [7]. However, the expression of c-FLIP and its relation to tumor cell apoptosis mediated by therapeutic agents remain largely elusive in PTCLs.

Histone deacetylases inhibitors (HDACIs) constitute a group of compounds that promote histone acetylation and transcription of genes involved in multiple cellular processes including apoptosis [8,9]. Several HDACIs have been proven effective in treating PTCLs. Recent studies showed that apoptosis induced by HDACIs in tumor cells is related to downregulation of c-FLIP and activation of TNF-related apoptosis-inducing ligand (TRAIL) signaling [10]. The mode of action of HDACIs on c-FLIP expression and extrinsic apoptosis needs to be further investigated in PTCLs.

Cellular transduction pathways play an important role on cancer cell response to treatment. NF-κB is a major signaling cascade involved in PTCLs, as revealed by gene expression profiling [11,12]. Constitutive activation of NF-κB causes chemoresistance of PTCLs but indicates tumor cell sensitivity to bio-therapeutic agent like proteasome inhibitor Bortezomib [13]. In the present study, we further addressed the clinical significance of NF-κB target gene *c-FLIP* in PTCLs, as well as the molecular mechanism of HDACIs on c-FLIP modulation and apoptosis induction in T-cell lymphoma both in vitro and in vivo. Functioned as an anti-apoptotic protein of extrinsic pathway, c-FLIP reflected tumor progression and resistance to chemotherapeutic agents, but could be targeted by HDAC1-mediated NF-κB inactivation and conferred T-lymphoma cell sensitivity to HDACIs.

## Results

### *c-FLIP* was overexpressed and related to tumor progression in PTCLs

**Figure 1:**
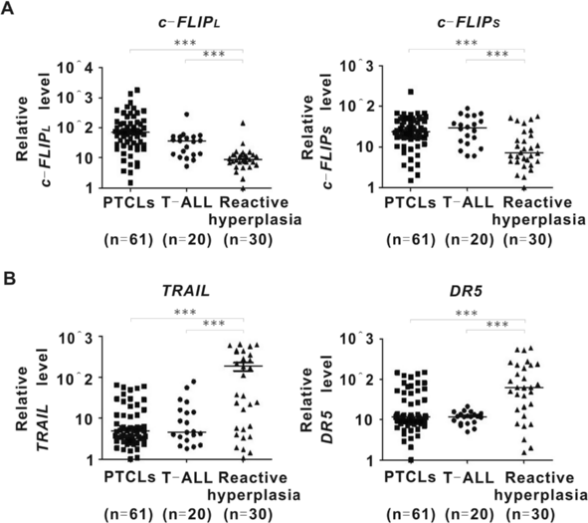
**c-FLIP was overexpressed and related to decreased TRAIL/DR5 expression in PTCLs patients.** Long and short isoform of *c-FLIP* gene (*c-FLIP*_*L*_ and *c-FLIP*_*S*_) **(A)**, as well as *TRAIL* and *DR5* expression **(B)** were detected by real-time PCR in PTCLs, T-ALL and reactive hyperplasia. ***, P < 0.001 comparing with reactive hyperplasia. All gene expression levels were calculated by ^ΔΔ^CT method based on the calibrator Jurkat cells.

Compared with reactive hyperplasia, long and short isoform of *c-FLIP* gene (*c-FLIP*_*L*_ and *c-FLIP*_*S*_) were overexpressed in patients with PTCLs and T-cell acute lymphoblastic leukemia (T-ALL) (P all <0.001, Figure 1A), in agreement with significant downregulation of extrinsic apoptosis-inducing signaling ligand *TRAIL* and its receptor *DR5* (P all <0.001, Figure 1B). Therefore, c-FLIP was potentially an indicator of defective extrinsic apoptosis in PTCLs.

Considering that *c-FLIP*_*L*_ was the main isoform expressed in PTCLs and did not vary from histological subtypes (Additional file 1: Figure S1), the relation of *c-FLIP*_*L*_ with clinical and biological parameters was studied. The median expression of *c-FLIP*_*L*_ in PTCLs was 70.06. The patients with *c-FLIP* expression level over and equal to the median value were regarded as high *c-FLIP* expression, whereas those below the median value were included in the low *c-FLIP* expression. Clinically, high *c-FLIP* expression was significantly associated with elevated serum lactate dehydrogenase (LDH) level and International Prognostic Index (IPI) indicating intermediate-high and high-risk (P = 0.036 and P = 0.010, respectively, Table 1).

**Table 1:** **Clinical and biological characteristics of PTCL patients (n = 61)**

	**High** ***c-FLIP***	**Low** ***c-FLIP***	**P-value**
Age			
> 60 years	11	12	0.291
≤ 60 years	19	19	
Sex			
Female	10	9	0.963
Male	20	22	
Ann Arbor stage			
I to II	14	13	0.257
III to IV	16	18	
Extranodal involvement			
No	19	25	0.091
Yes	11	6	
Serum LDH level			
Normal	11	19	0.036
Abnormal	19	12	
IPI score			
Low and intermediate low risk	16	25	0.010
Intermediate high and high risk	14	6	

*Abbreviations:* *LDH* lactate dehydrogenase, *IPI* International Prognostic Index.

### Molecular inhibition of c-FLIP sensitized T-lymphoma cells to chemotherapeutic agents

**Figure 2:**
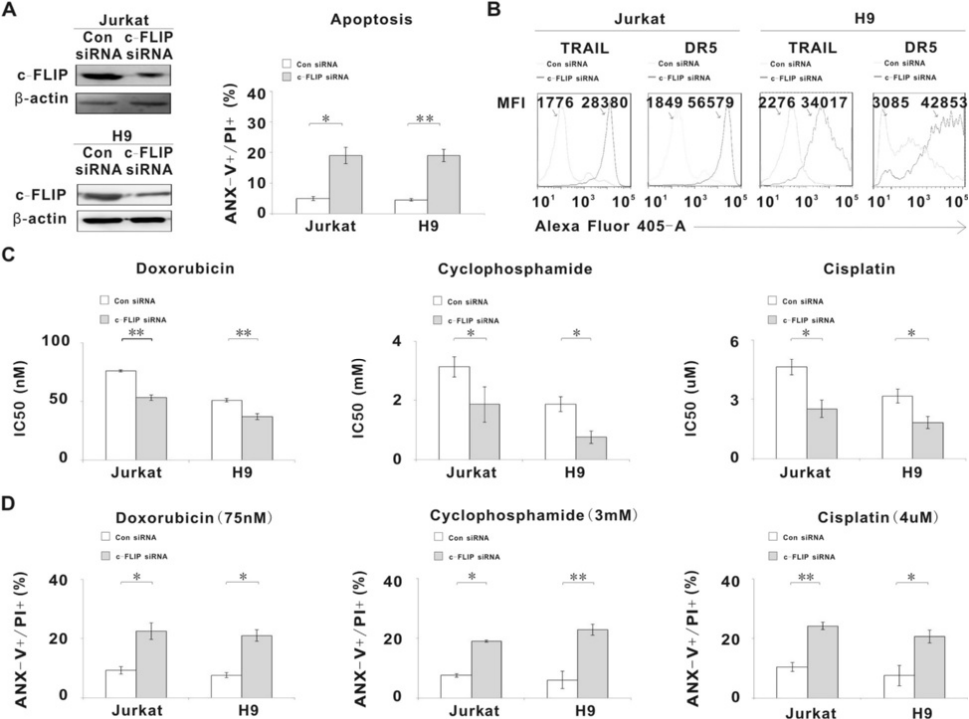
**Molecular inhibition of c-FLIP induced T-lymphoma cell apoptosis and sensitized tumor cells to chemotherapeutic agents. A**, c-FLIP expression of Jurkat and H9 cells transfected with Con siRNA or c-FLIP siRNA was confirmed by western blot (Left panel). Cell apoptosis of Jurkat and H9 cells transfected with Con siRNA or c-FLIP siRNA was measured by Annexin-V/PI assay (Right panel). **B**, TRAIL and DR5 expression of Jurkat and H9 cells transfected with Con siRNA or c-FLIP siRNA was assessed by flow cytometry. MFI, median fluorescence index. **C**, IC50 of doxorubicin, cyclophosphamide and cisplatin using Jurkat cells and H9 cells transfected with Con siRNA or c-FLIP siRNA were determined by MTT assay after 72 h incubation with chemotherapeutic agents. **D**, Cell apoptosis of Jurkat cells and H9 cells transfected with Con siRNA or c-FLIP siRNA was measured by Annexin-V/PI assay after treatment with doxorubicin (75 nm), cyclophosphamide (3 mM) and cisplatin (4 μM) for 72 h, respectively. *, P < 0.05, **, P < 0.01 comparing with the negative control (Con siRNA).

To better define the biological function of c-FLIP in PTCLs, Jurkat and H9 cells were transfected with specific c-FLIP small-interfering RNA (siRNA). The effect of c-FLIP siRNA on c-FLIP expression was confirmed by western blot (Figure 2A). Comparing with the control siRNA (Con siRNA), c-FLIP siRNA resulted in remarkable induction of tumor cell apoptosis (Figure 2A, P = 0.014 and P = 0.005, respectively), as well as increase of TRAIL and DR5 expression (Representative results shown in Figure 2B). Moreover, effects of treatment of both cells with chemotherapeutic agents such as doxorubicin, cyclophosphamide and cisplatin that are regularly applied to treat lymphoma, were analyzed by MTT assay and the concentration of the drug requires for 50% growth inhibition (IC50) was determined. The c-FLIP siRNA transfected cells were more sensitive to these agents than those transfected with the Con siRNA (P = 0.005, P = 0.015, P = 0.012 in Jurkat cells and P = 0.002, P = 0.049, P = 0.012 in H9 cells, respectively, Figure 2C), with corresponding increase of tumor cell apoptosis in c-FLIP siRNA group (P = 0.021, P = 0.014, P = 0.008 in Jurkat cells and P = 0.012, P = 0.005, P = 0.020 in H9 cells, respectively, Figure 2D). These data indicated that c-FLIP conferred tumor cell resistance to chemotherapy.

### HDACIs induced extrinsic apoptosis and inhibited c-FLIP expression of T-lymphoma cells

**Figure 3:**
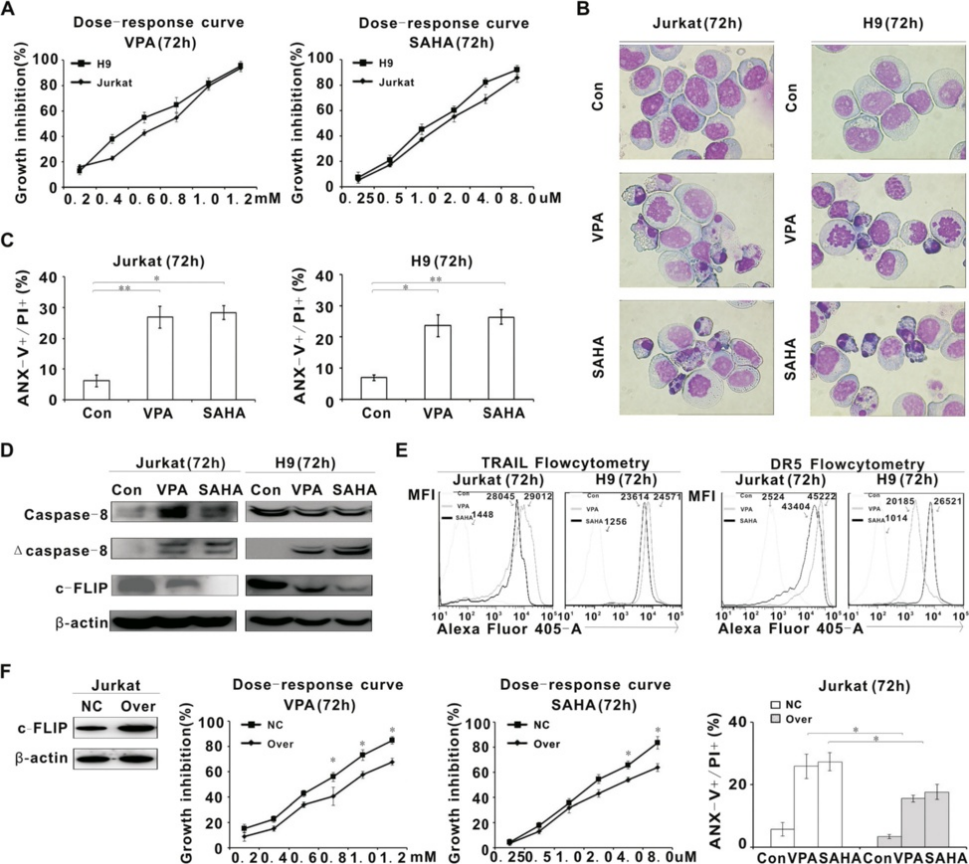
**HDACIs triggered extrinsic apoptosis of T-lymphoma cells. A**, Cell growth inhibition of Jurkat and H9 cells treated with different concentrations of HDACIs (VPA and SAHA) for 72 h was determined by MTT assay. **B**, Representative morphology of Jurkat and H9 cells treated with VPA (0.5 mM) and SAHA (2 μM) for 72 h was revealed by Wright’s staining. **C**, Cell apoptosis was measured by Annexin-V/PI assay in Jurkat and H9 cells treated with VPA (0.5 mM) and SAHA (2 μM) for 72 h. **D**, Caspase-8, cleaved caspase-8 and c-FLIP expression were detected by western blot. **E**, TRAIL and DR5 expression were detected by flow cytometry in Jurkat and H9 cells treated with VPA (0.5 mM) and SAHA (2 μM) for 72 h. **F**, c-FLIP expression of Jurkat cells transfected with negative control (NC) or c-FLIP-overexpressing vector (Over) was confirmed by western blot (Left panel). Cell growth inhibition of these cells treated with different concentrations of HDACIs (VPA and SAHA) for 72 h was determined by MTT assay (Middle panel). Cell apoptosis of these cells treated with VPA (0.5 mM) and SAHA (2 μM) for 72 h was measured by Annexin-V/PI assay (Right panel). *, P < 0.05 comparing with the negative control (NC).

To identify the possible role of bio-therapeutic agents HDACIs on c-FLIP, Jurkat and H9 cells were cultured with valproic acid (VPA) and suberoylanilide hydroxamic acid (SAHA). As shown in Figure 3A, HDACIs exerted substantial growth inhibition in both cells, which displayed characteristic morphological changes of apoptosis, such as shrinking cytoplasm, condensed chromatin and nuclear fragmentation with intact cell membrane (Figure 3B). Meanwhile, the percentage of Annexin V+/PI+ cells was significantly increased in HDACIs-treated cells (VPA, P = 0.002 and P = 0.023, SAHA, P = 0.013 and P = 0.003, respectively, Figure 3C).

HDACIs induced cleavage of Caspase-8, as well as decrease of c-FLIP expression both at the protein level (Figure 3D) and at the transcriptional level (Additional file 2: Figure S2A, VPA, P = 0.004 and P = 0.008, SAHA, P < 0.001 and P = 0.008, respectively). Meanwhile, significantly increased protein (Figure 3E) and gene expression of TRAIL and DR5 (Additional file 2: Figure S2B, TRAIL, VPA, P = 0.004 and P = 0.002, SAHA, P = 0.002 and P = 0.005; DR5, VPA, P = 0.001 and P = 0.012, SAHA, P = 0.001 and P = 0.009, respectively) were also observed upon HDACIs treatment, indicating that HDACI-induced extrinsic apoptosis was associated with downregulation of c-FLIP. In parallel to c-FLIP overexpression, T-lymphoma cells were less sensitive to VPA and SAHA, with decreased percentage of Annexin V+/PI+ cells (VPA, P = 0.031, SAHA, P = 0.018, respectively) (Figure 3F).

### HDACI-mediated c-FLIP downregulation was related to NF-κB inactivation via interrupting p50 interaction with c-FLIP

**Figure 4:**
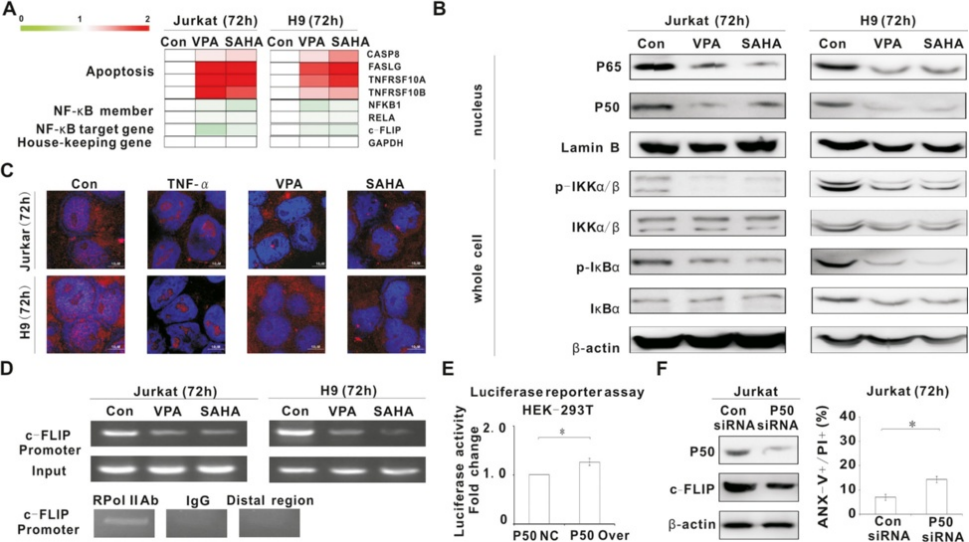
**HDACI-mediated c-FLIP downregulation was related to NF-κB inactivation via interrupting p50 interaction with c-FLIP. A**, Apoptosis and NF-κB-associated gene expression were detected in Jurkat and H9 cells treated with VPA (0.5 mM) and SAHA (2 μM) for 72 h. The genes were classified as extrinsic apoptosis-related genes, NF-κB members and NF-κB target gene *c-FLIP*. **B**, Nuclear P65 and P50 expression, as well as whole cell p-IKKαβ, IKKαβ and p-IκBα expression were detected in Jurkat and H9 cells treated with VPA (0.5 mM) and SAHA (2 μM) for 72 h. **C**, Nuclear P50 expression was evaluated in Jurkat and H9 cells pre-incubated with TNF-α (10 ng/ml) for 4 h and treated with VPA (0.5 mM) and SAHA (2 μM) for 72 h. **D**, The activity of P50 binding to the *c-FLIP* promoter was measured by CHIP assay in Jurkat and H9 cells treated with VPA (0.5 mM) and SAHA (2 μM) for 72 h. DNA-protein complexes from Jurkat and H9 cells were precipitated with anti-P50 antibody and amplified with primers for the region of the binding site using PCR (Upper panel). Antibody against RNA Polymerase II was referred as the positive control. Non-specific Ig and PCR with primers amplifying the distal region of the c-FLIP promoter were referred as the negative control (Lower panel). **E**, The effect of P50 on transcriptional activity of the c-FLIP promoter was measured by luciferase reporter assay in HEK-293T cells transfected with negative control (P50 NC) or P50-overexpressing vector (P50 Over). *, P < 0.05 comparing with the negative control (NC). **F**, P50 and c-FLIP expression were detected in Jurkat cells transfected with Con siRNA or P50 siRNA. Cell apoptosis of Jurkat cells transfected with Con siRNA or P50 siRNA was measured. *, P < 0.05 comparing with the negative control (Con siRNA).

*c-FLIP* is a target gene of NF-κB [14,15]. Extrinsic apoptosis-related genes, main NF-κB members and NF-κB target genes were assessed using Human NF-κB Signaling Pathway Plus PCR Array, before and after VPA or SAHA treatment (Figure 4A). In according with increased expression of apoptotic genes, NF-κB members (P65 and P50) and NF-κB target gene c-FLIP expression were decreased when treated with HDACIs. As revealed by western blot, nuclear P65 and P50 expression were downregulated, along with decrease of phosphorylated IKKα/β (p-IKKα/β) and phosphorylated IκBα (p-IκBα) expression in HDACI-treated cells (Figure 4B).

NF-κB member P50 is one of the regulators of c-FLIP [16]. As detected by immunofluorescence assay, VPA and SAHA blocked TNF-α-induced nuclear translocation of P50 in Jurkat and H9 cells (Figure 4C). Therefore, HDACIs could inhibit both intrinsic and extrinsic activation of P50. Chromatin immunoprecipitation (CHIP) assay was subsequently performed to explore the interaction of P50 with *c-FLIP* promoter region. The results showed that P50 was able to bind with the *c-FLIP* promoter and the binding activity declined after HDACIs treatment, as measured by PCR (Figure 4D). As shown by luciferase reporter assay, p50 activated the transcriptional activity of the *c-FLIP* promoter (P = 0.022, Figure 4E). To enforce the role of p50, Jurkat cells were transfected with specific P50 siRNA. The results showed that molecular silencing of p50 reduced the c-FLIP level (Figure 4F) and T lymphoma cells were less sensitive to apoptosis (P = 0.021, Figure 4F). Together, HDACI-induced c-FLIP downregulation was related to inactivation of NF-κB members, particularly P50.

### HDACIs inactivated P50 through inhibiting HDAC1

**Figure 5:**
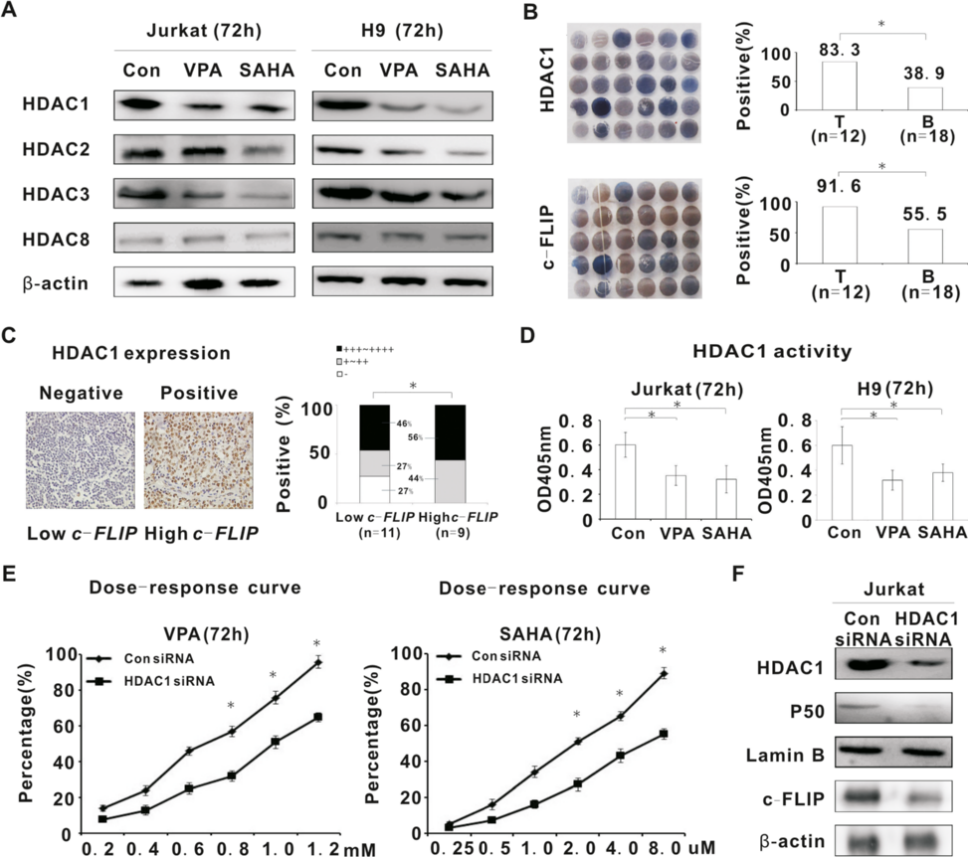
**HDACIs downregulated NF-κB signaling through HDAC1. A**, Expression of Class I HDACs including HDAC1, HDAC2, HDAC3 and HDAC8 were detected by western blot in Jurkat and H9 cells treated with VPA (0.5 mM) and SAHA (2 μM) for 72 h. **B**, HDAC1 and c-FLIP expression were determined by tissue array. *, P < 0.05 comparing with B-cell lymphomas. **C**, HDAC1 expression was evaluated by immunohistochemical study on tumor sections of PTCLs patients according to *c-FLIP* expression. The histogram represented the percentage of HDAC1-positive tumors. *, P < 0.05 comparing with low *c-FLIP* group. **D**, HDAC1 enzymatic activity was assessed by ELISA in Jurkat and H9 cells treated with VPA (0.5 mM) and SAHA (2 μM) for 72 h. *, P < 0.05 comparing with the untreated (Control) group. **E**, Cell growth inhibition of Jurkat cells transfected with Con siRNA or HDAC1 siRNA treated with different concentrations of HDACIs (VPA and SAHA) was determined by MTT assay. *, P < 0.05 comparing with the negative control (Con siRNA). **F**, HDAC1, P50 and c-FLIP expression were detected by western blot in Jurkat cells transfected with Con siRNA or HDAC1 siRNA.

As Class I HDACIs, VPA inhibited HDAC1 and HDAC3, whereas SAHA inhibited HDAC1, HDAC2 and HDAC3 expression (Figure 5A). Tissue array (Figure 5B) showed that HDAC1 was more frequently observed in T-cell lymphomas than in B-cell lymphomas (83.3% vs 38.9%, P = 0.016) and correlated with *c-FLIP* expression (r = 0.795). This was also present in tumor samples of patients with PTCLs (P = 0.035, Figure 5C). No significant difference of other members of Class I HDACIs (HDAC2, HDAC3, and HDAC8) was observed between T-cell lymphomas and B-cell lymphomas (data not shown).

Considering that HDAC1 was the main HDAC member involved in T-cell lymphomas, further study was focused on HDAC1 regulation by VPA and SAHA. In Jurkat and H9 cells, HDACIs-mediated HDAC1 downregulation was accompanied by reduced enzymatic activity of HDAC1 (VPA, P = 0.037 and P = 0.029, SAHA, P = 0.047 and P = 0.032, respectively, Figure 5D). Moreover, Jurkat cells were transfected with specific HDAC1 siRNA. Molecular silencing of HDAC1 significantly diminished HDACIs-mediated inhibition of tumor cell growth, consistent with decrease of P50 and c-FLIP expression (Figure 5E and 5F).

### HDACIs modulated HDAC1/P50/c-FLIP axis and induced in situ apoptosis in T-cell lymphoma in vivo

**Figure 6:**
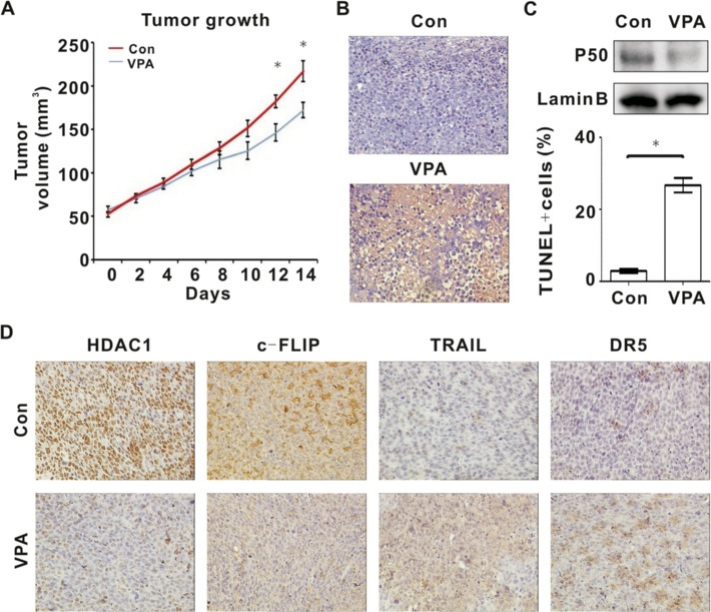
**VPA modulated HDAC1/P50/c-FLIP axis and induced tumor cell apoptosis in a murine xenograft T-lymphoma model. A**, The tumor growth curve of the Control group and the VPA group (0.4%w/v in the drinking water daily) in murine xenograft model established with subcutaneous injection of Jurkat cells. **B**, In situ apoptosis of the Control group and the VPA group was assessed by TUNEL assay. *, P < 0.05 comparing with the untreated (Control) group. **C**, P50 expression of the Control group and the VPA group was detected by western blot. **D**, HDAC1, c-FLIP, TRAIL and DR5 expression were evaluated by immunohistochemical study in the Control group and the VPA group.

The in vivo anti-tumor activity of HDACIs was further evaluated in a murine xenograft model of T-cell lymphoma. Subcutaneous inoculation of Jurkat cells into nude mice resulted in a tumor formation at the site of injection in all mice. The size of tumors formed in mice treated with oral VPA was significantly smaller than those of the untreated group since 12 days of treatment (Day 12, P = 0.049, Day 14, P = 0.012, respectively, Figure 6A).

To search for more evidence of tumor cell apoptosis, terminal deoxytransferase-catalyzed DNA nick-end labeling (TUNEL) assay was performed on mice tumor sections. Compared with the Control group, the number of the apoptotic tumor cells was significantly increased in the VPA group (P = 0.023, Figure 6B). In according with in vitro results, P50 (Figure 6C), HDAC1 and c-FLIP expression (Figure 6D) were decreased, while TRAIL and DR5 expression (Figure 6D) were increased upon VPA treatment.

## Discussion

Resistance to apoptosis becomes one of the hallmarks of human cancers [17]. In normal T-lymphocytes, *c-FLIP*_*L*_ and *c-FLIP*_*S*_ are expressed to protect T-cells from apoptosis [18]. In cutaneous T-cell lymphoma, c-FLIP upregulation inhibited TRAIL and DR5 expression, resulting in tumor cell resistance to extrinsic apoptosis [10,19]. Here we showed that *c-FLIP* is overexpressed in PTCLs and associated with decreased TRAIL/DR5 expression of the tumors and adverse clinical parameters of the patients. This further demonstrated that defective extrinsic apoptosis is essential for tumor progression of PTCLs and the biological behavior of malignant T-cells could successfully distinguish the clinical heterogeneity of this disease.

It is well recognized that tumor cells often survive from chemotherapy via acquiring resistance to apoptosis. Our experimental data confirmed that anti-apoptotic c-FLIP contributes to chemoresistance of T-lymphoma cells, since inhibition of c-FLIP triggered apoptosis and sensitized tumor cells to chemotherapeutic agents. Interestingly, difficult to be overcome by chemotherapy, c-FLIP expression was targeted by HDACIs, consistent with increase of TRAIL/DR5 expression and subsequent activation of extrinsic apoptosis. Induction of apoptosis by HDACI has been reported in cutaneous T-cell lymphoma and related to downregulation of c-FLIP and enhanced TRAIL signaling [10]. Therefore, our results advocated HDACIs to be potent agents for PTCLs, particularly chemoresistant tumors, and c-FLIP could be a potential biomarker to indicate lymphoma cell sensitivity to HDACIs.

Cell apoptosis is controlled by NF-κB, mainly as P65 and P50 [20]. P50 acts as a key regulator of NF-κB target gene *c-FLIP* [16]. In our study, HDACIs inhibited both intrinsic and extrinsic activity of P50, interrupted P50 interaction with *c-FLIP* promoter, and subsequently downregulated c-FLIP expression. As mechanism of action, HDACI could inactivate NF-κB pathway through interfering with recruitment of transcription factors to P50 promoter [21] or blocking IκBα degradation and subsequent nuclear NF-κB translocation [22]. Recent findings suggested that clinical efficiency of bio-therapeutic agent targeting NF-κB is independent on P65 expression in PTCLs patients [23]. Instead, P50 was shown to reflect tumor cell sensitivity to HDACIs [24]. As the determinant of c-FLIP expression [16], NF-κB member P50 is thus closely related to HDACIs-mediated apoptosis and c-FLIP downregulation in PTCLs.

Increased Class I HDAC activity and expression are associated with NF-κB activation of tumor cells [25]. As the major HDAC in PTCLs [26], both protein expression and enzymatic activity of HDAC1 were reduced upon treatment with Class I HDACIs like VPA and SAHA [27,28]. Molecular silencing of HDAC1 by siRNA mimicked the effect of HDACIs on tumor cell growth, as well as on P50 and c-FLIP downregulation. Therefore, HDAC1 could function as the action site of VPA and SAHA in PTCLs. It is previously reported that in acute myeloid leukemia [16], HDAC1 is displaced from P50 homodimers bound to anti-apoptotic genes, contributing to NF-κB inactivation and c-FLIP downregulation. In glioblastoma, another transcription factor c-myc could be recruited on *c-FLIP* promoter upon HDACI treatment and resulted in decreased expression of c-FLIP [29]. Our study showed that VPA and SAHA induced lymphoma cell apoptosis through inactivating HDAC1/P50/c-FLIP axis, indicative an alternative mechanism of HDACIs on NF-κB activation and tumor cell apoptosis in PTCLs.

c-FLIP inhibited extrinsic apoptosis and favored tumor progression, providing a therapeutic target in PTCLs. HDACIs are convictive as apoptosis-inducing agents in PTCLs, either alone or combined with other agents [9,30,31]. HDAC1/P50/c-FLIP axis could thus be helpful to determine clinical efficiency of HDACIs.

## Conclusions

Our findings highlighted that c-FLIP was involved in defective apoptosis, tumor progression in PTCLs and related to T-lymphoma cell apoptosis induced by HDACIs through inhibiting HDAC1/P50/c-FLIP axis.

## Methods

### Patients

Sixty-one patients diagnosed as PTCLs were enrolled in this study, including PTCL-not otherwise specified (33 cases), angioimmunoblastic T-cell lymphoma (16 cases), and anaplastic large-cell lymphoma (12 cases). Histologic diagnoses were established according to the World Health Organization classification [32]. Induction chemotherapy consisted of 6 to 8 cycles of CHOP or CHOP-like regimen. The clinical and biological features of the patients were listed in Table 1. Twenty cases with T-ALL and thirty cases with reactive hyperplasia were referred as controls. The study was approved by the Shanghai Rui Jin Hospital Review Board with informed consent obtained from all patients in accordance with the Declaration of Helsinki.

### Cells and reagents

T lymphoma cell lines (Jurkat and H9) and HEK-293 T cells were obtained from American Type Culture Collection (Manassas, VA, USA). Cells were cultured in RPMI-1640 medium supplemented with 10% heat-inactivated fetal bovine serum in humidified atmosphere of 95% air and 5% CO_2_ at 37°C. VPA was from Sigma-Aldrich (St Louis, MO, USA). SAHA was from Merck & Co (Darmstadt, Germany). Antibodies against Caspase-8, p-IKKα/β, p-IκBα and HDAC3 were purchased from Cell Signaling (Beverly, MA, USA). Antibodies against c-FLIP, P65, P50, IKKα/β, IκBα, HDAC1, HDAC2, HDAC8 and Lamin B were from Abcam (Cambridge, UK). Anti-β-actin antibody was from Sigma-Aldrich. Horseradish peroxidase-conjugated goat anti-mouse IgG and goat anti-rabbit IgG were from Santa Cruz Biotechnology (Santa Cruz, CA, USA).

### Cell proliferation and morphology

Cell proliferation was assessed by MTT assay and the absorbance was measured at 490 nm by spectrophotometry. Cell morphology was evaluated by Wright’s staining under light microscopy.

### Flow cytometric assay

Cell apoptosis was assessed using Annexin V-FITC Apoptosis Kits (Becton Dickinson, Franklin Lakes, NJ, USA) according to the manufacturer’s instructions. Expression levels of TRAIL and DR5 were quantified using antibodies against TRAIL (Cell signaling) and DR5 (Abnova, Walnut, CA, USA) as the primary antibodies, and DyLight 405 labeled anti-rabbit antibody (KPL, Washington DC, USA) as the secondary antibody. The median fluorescent intensity (MFI) was measured by flow cytometry.

### Real-time polymerase chain reaction (PCR)

Total RNA was extracted from frozen tissue of PTCLs and reactive hyperplasia, as well as bone marrow blasts of T-ALL, using TRIzol reagent (Invitrogen). cDNA was synthesized using PrimeScript RT reagent Kits with gDNA Eraser (TaKaRa, CA, USA) following the manufacturer’s instructions. Real-time polymerase chain reaction (PCR) was performed using SYBR Premix Ex Taq™ II (TaKaRa) on ABI Prism 7500 (Applied Biosystems, Bedford, MA, USA). The relative gene expression levels were calculated using the SDS2.4 software. The primer sequences were as follows: *c-FLIP*_*L*_ forward, 5′-ATTGCATTGGCAATGAGACAGAGC-3′; reverse, 5′-TCGGTGCTCGGGCATACAGG-3′, *c-FLIP*_*S*_ forward, 5′-ACCCTCACCTTGTTTCGGACTAT-3′; reverse, 5′-TGAGGACACATCAGATTTATCCAAA-3′, *TRAIL* forward, 5′-TCAGCACTTCAGGATGATGG-3′; reverse, 5′-CACCAGCTGTTTGGTTCTCA-3′, *DR5* forward, 5′-TGACGGGGAAGAGGAACTGA-3′; reverse, 5′-GGCTTTGACCATTTGGATTTGA-3′, *GAPDH* forward, 5′-GAAGGTGAAGGTCGGAGTC-3′; reverse, 5′-GAAGATGGTGATGGGATTTC-3′.

Real-time PCR of NF-κB signaling pathway was performed using the RT^2^ profiler PCR Array-Human NF-κB signaling pathway (QIAGEN Sciences, Frederick, MD, USA). GAPDH was used as the endogenous control and Jurkat cells for calibration. A relative quantification was calculated using the ^ΔΔ^CT method.

### Tissue array

A human lymphoma tissue array was purchased from US Biomax, Inc. (Rockville, MD, USA) containing 12 T-cell lymphomas and 18 B-cell lymphomas. Levels of protein expression were graded according to staining intensity (SI) and distribution using the immunoreactive score (IRS). IRS = SI × PP (percentage of positive cells). SI was defined as 0 = negative; 1 = weak; 2 = moderate; and 3 = strong. PP was scored as 1, <25%; 2, 25-50%; 3, 50-75%; and 4, 75-100% positive cells. IRS > 4 was determined as positive.

### Immunohistochemistry and immunofluorescence assay

Immunohistochemistry was performed on 5 μm-paraffin sections with an indirect immunoperoxidase method using the primary antibody against c-FLIP (1:200), TRAIL (1:800), DR5 (1:200), HDAC1 (1:100), HDAC2 (1:100), HDAC3 (1:100) and HDAC8 (1:100). Immunofluorescence assay was performed on methanol-fixed cells using P50 as the primary antibody and diaminotriazinylaminofluorescein-labeled donkey anti-rabbit-IgG antibody (Abcam) as the second antibody.

### Cell transfection

Jurkat cells were transfected with c-FLIP, P50 and HDAC1 siGENOME SMARTpool or Non-Targetingpool (Dharmacon) as the negative control using DharmaFECT2 transfection reagent (Dharmacon) following the manufacturer’s instruction. As for overexpression assay, Jurkat cells were transfected with c-FLIP-overexpressing vector or a control vector, electroporated at 250 V 25 ms in 4-mm cuvettes using a BTX ECM 830 and replated in fresh medium for further experiments.

### Nuclear and cytosolic fractionation

Cells were suspended in 400 μL lysis buffer (10 mM HEPES, 10 mM KCl, 1.5 mM MgCl_2_, 0.5 mM DTT, pH 7.9) with 0.2% Nonidet P-40 and protease inhibitor cocktail for 1 min on ice. After centrifuged for 1 min at 2500 × *g*, the supernatants were collected as cytoplasmic protein extracts. The pellets were washed with lysis buffer without Nonidet P-40, then re-suspended in 150 μL extraction buffer (20 mM HEPES, pH 7.9, 420 mM NaCl, 0.5 mM DTT, 0.2 mM EDTA and 25% glycerol), and incubated for 20 min on ice. After centrifuged at 12000 × *g* for 10 min, the supernatants were collected as nuclear protein extracts.

### Western blot

Cells were collected and lysed in 200 μL lysis buffer (Sigma Aldrich). Protein lysates (20 μg) were electrophoresed on 10% sodium dodecyl sulfate polyacrylamide gels and transferred to nitrocellulose membranes. Membranes were blocked with 5% non-fat dried milk and incubated overnight at 4°C with appropriate primary antibody, followed by horseradish peroxidase-linked secondary antibody. The immunocomplexes were visualized using chemiluminescence phototope-horseradish peroxidase Kits. Lamin B and β-actin were used to ensure equivalent loading of nuclear and whole cell protein, respectively.

### CHIP assay

CHIP assay was performed using EZ-ChIP Kits (Millipore, Billerica, MA, USA) to identify the interaction between DNA and protein following the manufacturer’s instructions. Antibody against RNA Polymerase II was referred as the positive control and non-specific Ig and PCR with primers amplifying the distal region of the c-FLIP promoter as the negative control (forward, 5′-CCCGGGTTCAAGCAATTCTC-3′; reverse, 5′-GGATCACGAGGTCAGGAGTT-3′).

### Luciferase report assay

HEK-293T cells were transfected with luciferase reporter and P50 overexpressing vector, using Lipofectamine 2000 (Invitrogen) according to the manufacturer’s instructions. Protein was collected 24 h after transfection, using the Passive Lysis Buffer (30 μL per well) provided as part of the Dual-Luciferase Reporter Assay System kit (Promega). Firefly and Renilla luciferase activities were examined by the Dual-Luciferase Reporter Assay System and detected by a Centro XS3 LB960 Luminometer (Berthold).

### Enzyme-linked immunosorbent assay

Enzymatic activity of HDAC1 was quantified by enzyme-linked immunosorbent assay using HDAC colorimetric Kit (BioVision, Milpitas, CA, USA) according to the manufacturer’s instructions.

### TUNEL assay

In situ cell apoptosis was determined by detection of fragmented DNA, using DeadEnd Colorimetric Terminal deoxytransferase-catalyzed DNA nick-end labeling System (Promega Corporation, Madison, WI, USA), on 5 μm-paraffin sections according to the manufacturer’s instructions.

### Murine model

To test the in vivo efficiency of VPA, nude mice (5-6-week-old, obtained from Shanghai Laboratory Animal Center, Shanghai, China) were injected with 4 × 10^7^ Jurkat cells into the right flank. Treatments started after tumor became about 0.5 cm × 0.5 cm in surface (Day 0). The control group received saline, the treatment group received oral VPA for 14 days (0.4%w/v in the drinking water daily).

### Statistical analysis

Difference of *c-FLIP* expression among groups were calculated using Mann–Whitney U test. The association between *c-FLIP* expression and clinical parameters was analyzed by Chisquare test. In vitro experimental results were expressed as mean±s.d. of data obtained from three separate experiments and determined by t-test to compare variance. P<0.05 was considered to be significant. Statistical analyses were performed on SPSS13.0 software.

## Additional files

Additional file 1: Figure S1. c-FLIP expression according to histologic subtypes of PTCLs. Elevated *c-FLIP* levels were observed among all the PTCL subtypes studied, including PTCL-not otherwise specified (PTCL-NOS), angioimmunoblastic T-cell lymphoma (AITCL), and anaplastic large-cell lymphoma (ALCL). Additional file 2: Figure S2. Extrinsic apoptotic gene expressions during HDACIs treatment in T-lymphoma cells. The expression of *c-FLIP* (A), *TRAIL* and *DR5* (B) were detected by real-time PCR in Jurkat and H9 cells treated with VPA (0.5 mM) and SAHA (2 μM) for 72 h. ***, P < 0.001, **, P < 0.01, comparing with the untreated (Control) group.

## Abbreviations

PTCLs: Peripheral T-cell lymphomas
HDACIs: Histone deacetylase inhibitors
VPA: Valproic acid
SAHA: Suberoylanilide hydroxamic acid
c-FLIP: Cellular FLICE inhibitory protein
TRAIL: TNF-related apoptosis-inducing ligand
T-ALL: T-cell acute lymphoblastic leukemia
LDH: Serum lactate dehydrogenase
IPI: International prognostic index
siRNA: Small-interfering RNA
CHIP: Chromatin immunoprecipitation
TUNEL: Terminal deoxytransferase-catalyzed DNA nick-end labeling

## References

[1] Campo E, Swerdlow SH, Harris NL, Pileri S, Stein H, Jaffe ES (2011). The 2008 WHO classification of lymphoid neoplasms and beyond: evolving concepts and practical applications. Blood.

[2] Vose J, Armitage J, Weisenburger D (2008). International peripheral T-cell and natural killer/T-cell lymphoma study: pathology findings and clinical outcomes. J Clin Oncol.

[3] Johnstone RW, Ruefli AA, Lowe SW (2002). Apoptosis: a link between cancer genetics and chemotherapy. Cell.

[4] Reed JC, Pellecchia M (2005). Apoptosis-based therapies for hematologic malignancies. Blood.

[5] Shaffer AL, Rosenwald A, Staudt LM (2002). Lymphoid malignancies: the dark side of B-cell differentiation. Nat Rev Immunol.

[6] Fulda S (2013). Targeting c-FLICE-like inhibitory protein (CFLAR) in cancer. Expert Opin Ther Targets.

[7] Valente G, Manfroi F, Peracchio C, Nicotra G, Castino R, Nicosia G, Kerim S, Isidoro C (2006). cFLIP expression correlates with tumour progression and patient outcome in non-Hodgkin lymphomas of low grade of malignancy. Br J Haematol.

[8] Marks P, Rifkind RA, Richon VM, Breslow R, Miller T, Kelly WK (2001). Histone deacetylases and cancer: causes and therapies. Nat Rev Cancer.

[9] Reimer P, Chawla S (2013). Long-term complete remission with belinostat in a patient with chemotherapy refractory peripheral T-cell lymphoma. J Hematol Oncol.

[10] Al-Yacoub N, Fecker LF, Mobs M, Plotz M, Braun FK, Sterry W, Eberle J (2012). Apoptosis induction by SAHA in cutaneous T-cell lymphoma cells is related to downregulation of c-FLIP and enhanced TRAIL signaling. J Investig Dermatol.

[11] Odqvist L, Sanchez-Beato M, Montes-Moreno S, Martin-Sanchez E, Pajares R, Sanchez-Verde L, Ortiz-Romero PL, Rodriguez J, Rodriguez-Pinilla SM, Iniesta-Martinez F, Solera-Arroyo JC, Ramos-Asensio R, Flores T, Palanca JM, Bragado FG, Franjo PD, Piris MA (2013). NIK controls classical and alternative NF-kappaB activation and is necessary for the survival of human T-cell lymphoma cells. Clin Cancer Res.

[12] Pileri SA, Piccaluga PP (2012). New molecular insights into peripheral T cell lymphomas. J Clin Investig.

[13] Zhang QL, Wang L, Zhang YW, Jiang XX, Yang F, Wu WL, Janin A, Chen Z, Shen ZX, Chen SJ, Zhao WL (2009). The proteasome inhibitor bortezomib interacts synergistically with the histone deacetylase inhibitor suberoylanilide hydroxamic acid to induce T-leukemia/lymphoma cells apoptosis. Leukemia.

[14] Irmler M, Thome M, Hahne M, Schneider P, Hofmann K, Steiner V, Bodmer JL, Schroter M, Burns K, Mattmann C, Rimoldi D, French LE, Tschopp J (1997). Inhibition of death receptor signals by cellular FLIP. Nature.

[15] McLornan D, Hay J, McLaughlin K, Holohan C, Burnett AK, Hills RK, Johnston PG, Mills KI, McMullin MF, Longley DB, Gilkes A (2013). Prognostic and therapeutic relevance of c-FLIP in acute myeloid leukaemia. Br J Haematol.

[16] Paz-Priel I, Houng S, Dooher J, Friedman AD (2011). C/EBPalpha and C/EBPalpha oncoproteins regulate nfkb1 and displace histone deacetylases from NF-kappaB p50 homodimers to induce NF-kappaB target genes. Blood.

[17] Hanahan D, Weinberg RA (2011). Hallmarks of cancer: the next generation. Cell.

[18] Zhang N, Hopkins K, He YW (2008). c-FLIP protects mature T lymphocytes from TCR-mediated killing. J Immunol.

[19] Martin-Perez R, Niwa M, Lopez-Rivas A (2012). ER stress sensitizes cells to TRAIL through down-regulation of FLIP and Mcl-1 and PERK-dependent up-regulation of TRAIL-R2. Apoptosis.

[20] Dolcet X, Llobet D, Pallares J, Matias-Guiu X (2005). NF-kB in development and progression of human cancer. Virchows Arch.

[21] Furumai R, Ito A, Ogawa K, Maeda S, Saito A, Nishino N, Horinouchi S, Yoshida M (2011). Histone deacetylase inhibitors block nuclear factor-kappaB-dependent transcription by interfering with RNA polymerase II recruitment. Cancer Sci.

[22] Zhong HM, Ding QH, Chen WP, Luo RB (2013). Vorinostat, a HDAC inhibitor, showed anti-osteoarthritic activities through inhibition of iNOS and MMP expression, p38 and ERK phosphorylation and blocking NF-kappaB nuclear translocation. Int Immunopharmacol.

[23] Kim SJ, Yoon DH, Kang HJ, Kim JS, Park SK, Kim HJ, Lee J, Ryoo BY, Ko YH, Huh J, Yang WI, Kim HK, Min SK, Lee SS, Do IG, Suh C, Kim WS (2012). Bortezomib in combination with CHOP as first-line treatment for patients with stage III/IV peripheral T-cell lymphomas: a multicentre, single-arm, phase 2 trial. Eur J Cancer.

[24] Kubo M, Kanaya N, Petrossian K, Ye J, Warden C, Liu Z, Nishimura R, Osako T, Okido M, Shimada K, Takahashi M, Chu P, Yuan YC, Chen S (2013). Inhibition of the proliferation of acquired aromatase inhibitor-resistant breast cancer cells by histone deacetylase inhibitor LBH589 (panobinostat). Breast Cancer Res Treat.

[25] Lehmann A, Denkert C, Budczies J, Buckendahl AC, Darb-Esfahani S, Noske A, Muller BM, Bahra M, Neuhaus P, Dietel M, Kristiansen G, Weichert W (2009). High class I HDAC activity and expression are associated with RelA/p65 activation in pancreatic cancer in vitro and in vivo. BMC Cancer.

[26] Marquard L, Poulsen CB, Gjerdrum LM, de Nully BP, Christensen IJ, Jensen PB, Sehested M, Johansen P, Ralfkiaer E (2009). Histone deacetylase 1, 2, 6 and acetylated histone H4 in B- and T-cell lymphomas. Histopathology.

[27] Witt O, Deubzer HE, Milde T, Oehme I (2009). HDAC family: what are the cancer relevant targets?. Cancer Lett.

[28] Marks PA, Xu WS (2009). Histone deacetylase inhibitors: potential in cancer therapy. J Cell Biochem.

[29] Bangert A, Cristofanon S, Eckhardt I, Abhari BA, Kolodziej S, Hacker S, Vellanki SH, Lausen J, Debatin KM, Fulda S (2012). Histone deacetylase inhibitors sensitize glioblastoma cells to TRAIL-induced apoptosis by c-myc-mediated downregulation of cFLIP. Oncogene.

[30] Coiffier B, Pro B, Prince HM, Foss F, Sokol L, Greenwood M, Caballero D, Morschhauser F, Wilhelm M, Pinter-Brown L, Padmanabhan Iyer S, Shustov A, Nielsen T, Nichols J, Wolfson J, Balser B, Horwitz S (2014). Romidepsin for the treatment of relapsed/refractory peripheral T-cell lymphoma: pivotal study update demonstrates durable responses. J Hematol Oncol.

[31] Jain S, Zain J, O’Connor O (2012). Novel therapeutic agents for cutaneous T-cell lymphoma. J Hematol Oncol.

[32] Jaffe ES: **The 2008 WHO classification of lymphomas: implications for clinical practice and translational research.** *Hematology Am Soc Hematol Educ Program* 2009, 523–531. 10.1182/asheducation-2009.1.523 PMC6324557 20008237

