# Supplementary figures and images for: c-FLIP is involved in tumor progression of peripheral T-cell lymphoma and targeted by histone deacetylase inhibitors
Source: J Hematol Oncol. 2014 Dec 5;7:88. doi: 10.1186/s13045-014-0088-y (PMC4261569; doi:10.1186/s13045-014-0088-y)

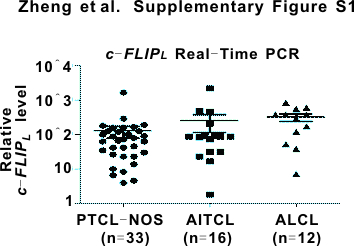

Supplement: Additional file 1: Figure S1. — c-FLIP expression according to histologic subtypes of PTCLs. Elevated c-FLIP levels were observed among all the PTCL subtypes studied, including PTCL-not otherwise specified (PTCL-NOS), angioimmunoblastic T-cell lymphoma (AITCL), and anaplastic large-cell lymphoma (ALCL). [file 13045_2014_88_MOESM1_ESM.jpeg]

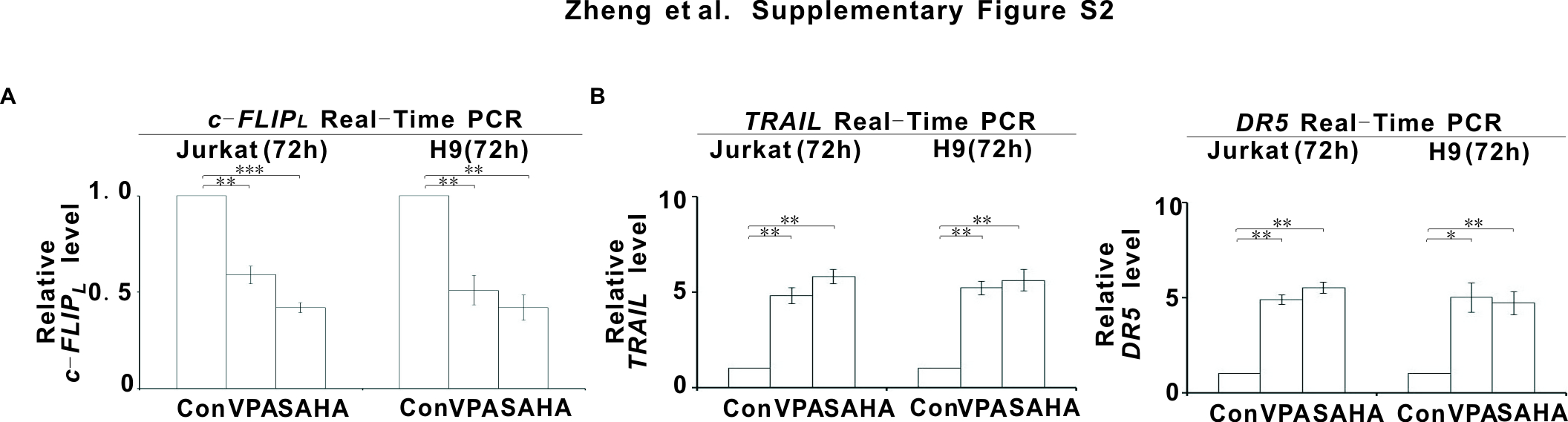

Supplement: Additional file 2: Figure S2. — Extrinsic apoptotic gene expressions during HDACIs treatment in T-lymphoma cells. The expression of c-FLIP (A), TRAIL and DR5 (B) were detected by real-time PCR in Jurkat and H9 cells treated with VPA (0.5 mM) and SAHA (2 μM) for 72 h. ***, P < 0.001, **, P < 0.01, comparing with the untreated (Control) group. [file 13045_2014_88_MOESM2_ESM.jpeg]
